# SIGMA: self-supervised inference of gene networks via masked auto-encoding

**DOI:** 10.3389/fgene.2026.1825728

**Published:** 2026-06-05

**Authors:** Qian Wang, Ziyi Zhang, Nan-Qing Liao, Shibin Yang, Zehua He

**Affiliations:** 1 School of Medicine, Guangxi University, Nanning, China; 2 Department of Pulmonary and Critical Care Medicine, The First Affiliated Hospital of Guangxi Medical University, Nanning, Guangxi, China; 3 Department of Plastic Surgery and Burns, Affiliated Hospital of Zunyi Medical University, Zunyi, China; 4 College of Life Science and Technology, Guangxi University, Nanning, China; 5 Department of Gastrointestinal Surgery, The First Affiliated Hospital, Sun Yat-sen University, Guangzhou, China; 6 Department of General Surgery, Guangxi Hospital Division of the First Affiliated Hospital of Sun Yat-sen University, Nanning, China

**Keywords:** expression profiles, gene regulatory networks, masked auto-encoder, self-supervised learning, transformer

## Abstract

**Objectives:**

Inferring gene regulatory networks (GRNs) from expression profiles is essential for identifying critical genes within complex disease pathways. However, current machine learning-based GRN inference methods face two challenges. Unsupervised methods struggle to achieve satisfactory accuracy in inference, while supervised methods are limited by the scarcity of high-quality interaction labels. Further, existing models demonstrate significant shortcomings when it comes to transferring reasoning to other GRN task subtypes. These issues affect GRN inference and hinder the ability to discover new regulatory patterns.

**Findings:**

To address these challenges, we have developed SIGMA: a transformer-based framework that uses self-supervised learning to pretrain the encoder on expression profiles. This alleviates the need for high-quality labels. During pretraining, it converts gene expression pairs into non-overlapping patches, and randomly masks some of these patches. This forces the encoder to extract correlation representations from the unmasked patches without label guidance, enabling the decoder to reconstruct the masked patches while preserving their similarity. Experiments have demonstrated that the pretrained encoder can accurately infer GRNs and be used to infer other subtypes, thereby reducing reliance on labels. Benchmark tests on human and mouse datasets have shown that SIGMA outperforms state-of-the-art methods. When applied to breast cancer datasets, SIGMA produced predictions that were consistent with established networks and identified candidate interactions that were not present in the gold-standard networks. Further investigation and experimental validation of these relationships is warranted.

## 1 Introduction

Gene regulatory networks (GRNs) are complex systems that define the correlations between transcription factors (TFs) and their direct target genes, specifically governing the transcriptional activation or repression of target genes mediated by TFs (Unger Avila et al., 2024). GRN inference is closely linked to understanding molecular mechanisms, target identification, and drug design, and gene expression profiles—a groundbreaking innovation in molecular biology—provide insights into the origin and dynamics of gene interactions (Lan et al., 2024b). However, previous GRN inference primarily relies on the experimental validation of each TF-target relationship, which has several limitations: it requires substantial cost and time investment and is susceptible to individual and environmental variations (Kim et al., 2023), significantly limiting its effectiveness in GRN inference tasks and necessitating more efficient methods.

Advances in machine learning have opened new avenues for GRN inference. These methods typically leverage gene expression profiles to identify interaction patterns and infer potential gene-gene relationships, treating experimentally confirmed interactions as “1” and unconfirmed ones as “0” to frame GRN inference as a binary classification problem (Zhao et al., 2021). They encompass both unsupervised and supervised learning approaches. Unsupervised methods infer GRNs by solely analyzing expression profiles without prior labels (Bellot et al., 2019; Li et al., 2024); methods such as GENIE3 (Huynh-Thu et al., 2010), PIDC (Chan et al., 2017), SCENIC (Aibar et al., 2017), and GRNBoost2 (Moerman et al., 2019) identify potential TF-target pair interactions by assessing correlation importance. However, the absence of prior labels leads to excessive identified correlations, preventing the distinction of genuine regulatory interactions and greatly restricting their application.

In contrast, supervised methods rely on prior labels to infer GRNs from expression profiles and often produce reliable results. Methods such as DeepSEM (Shu et al., 2021), CNNC (Yuan and Bar-Joseph, 2019), DGRNS (Zhao et al., 2022), and STGRN (Xu et al., 2023) transform TF-target pairs into feature matrices, using convolutional neural networks (CNNs), recurrent neural networks (RNNs), or transformer models (Vaswani et al., 2017) to accurately infer GRNs based on known gene relationships (Ji et al., 2024; Lan et al., 2024a). However, the distinction between “confirmed” and “unconfirmed” interactions is not always clear—these real but unconfirmed interactions hinder model inference and diminish its capabilities. Furthermore, supervised methods typically train the entire model on existing labels; new confirmed interactions require full retraining to adapt to updated GRNs, and discovering different GRN types often necessitates training new models from scratch, leading to substantial redundancy and waste. This leads us to ask: how can we train a more general and robust model that can be updated by adjusting only a few parameters and can infer multiple types of GRNs?

We attempted to answer this question from the following perspectives:

- GRN inference has been limited by the scarcity of labels. Unsupervised methods circumvent this requirement but underperform, whereas supervised methods achieve superior accuracy but require extensive high-quality labels. Self-supervised learning reduces reliance on label quality by using pretext tasks (He et al., 2020). Models extract intrinsic correlation representations (i.e., features) directly from raw data, bypassing manual annotation entirely. These universal features can be transferred seamlessly to diverse downstream tasks, delivering performance that matches or exceeds that of fully supervised baselines.
- The modeling of TF-target correlations does not require extensive preprocessing; gene expression sequences represent state changes and carry high information density. Although exploring the correlation of TF-target pairs through coefficients or co-expression histograms seems feasible, this type of preprocessing distorts the original semantics and introduces biases that hinder model inference. To preserve raw information while learning meaningful features, we use a modified masked auto-encoder (MAE) (He et al., 2022) pretext task: we mask portions of TF-target sequences and task the model with reconstructing them from unmasked segments while enforcing consistency with the original data. This approach preserves raw information, enables the model to capture intrinsic correlations, and can transfer across diverse GRN subtypes.
- Prior approaches typically correlate entire gene expression sequences; however, gene regulation is not a global process and emerges only during specific time windows. Forcing global connections often degrades model performance, a principle that models such as DGRNS (Zhao et al., 2022) and STGRN (Xu et al., 2023) have clearly validated. Following this insight, our approach not only significantly reduces the computational cost and memory usage, but also increases the information density of masked semantic units, enabling the model to learn more discriminative features.

Based on these analyses, we propose a transformer-based framework, Self-supervised Inference of GRNs via Masked Auto-encoding (SIGMA), inspired by MAE. SIGMA uses self-supervised learning to pretrain on unlabeled expression profiles. The framework transforms TF-target pairs into non-overlapping patches by randomly masking a portion of them. Through pretext tasks, the encoder extracts correlation representations from unmasked patches. This enables the decoder to reconstruct the masked patches and ensures representational similarity between the masked and reconstructed patches. Subsequent experiments revealed that a mask ratio of 70% enables the encoder to capture the optimal correlation representations. The pretrained encoder can accurately infer GRNs and facilitate the deep exploration of various subtypes. We assessed SIGMA’s performance on seven benchmark expression profile datasets from five cell lines, including two human and three mouse cell lines, spanning four distinct ground truth networks. Our findings demonstrate that SIGMA outperforms state-of-the-art methods, establishing a new approach for GRN inference based on self-supervised learning.

The contributions of the proposed SIGMA model are as follows:

- We have developed a powerful self-supervised learning framework for gene expression profiles. This framework improves the identification of intrinsic interactions by using pretext tasks to pretrain the encoder. This eliminates the need for prior labeling and optimizes the discovery of correlations.
- The effective combination of two pretraining strategies enables the precise extraction of correlation representations.
- SIGMA exhibits outstanding inferential performance across varied subtypes, and its robustness and generalization capabilities have been confirmed. SIGMA is the first self-supervised regression model developed for GRN inference.

## 2 Methods

**FIGURE 1:**
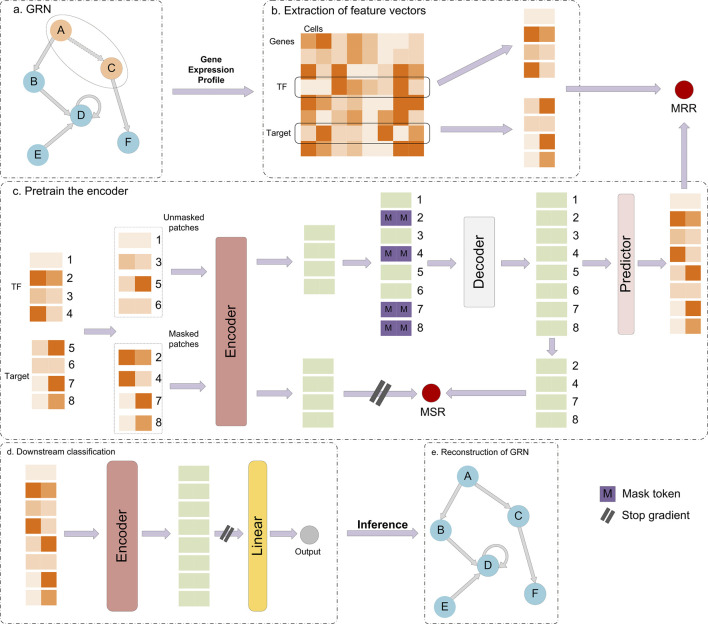
The SIGMA framework employs a structured methodology to infer GRN: **(a)** Initially, the framework retrieves known regulatory interactions from a database to serve as ground truth for the GRN inference process. **(b)** The gene expression data is processed to generate feature vectors for each potential TF-target gene pair. This preprocessing step involves the use of patches to capture relevant molecular interactions. **(c)** These feature vectors are input into the proposed pretrained model, which randomly separates them into “masked patches” and “unmasked patches.” Model training is conducted through two interrelated tasks: mask representation regression and enhancement of subvector similarity within the masked patches. **(d)** Following pretraining, the model’s encoder component is isolated for the specific task of regulatory network inference. The encoder is fed patches corresponding to the TF-target pairs, allowing it to interpret their relationships. Our experimental results indicate that retraining the pretrained encoder is not necessary; instead, only the final linear layer requires further training to enable. **(e)** GRN reconstruction.

Our approach is driven by the significant gaps present in gene interaction labels. During supervised model training, classifying “unidentified interactions” and “no interactions” under the same label would hinders the model’s inferencing capabilities. Conversely, unsupervised methods tend to explore correlations between genes without discerning whether these relationships are genuine regulatory interactions, as they lack label guidance. To achieve precise GRN inference, and alleviate dependence on high-quality labels, we propose SIGMA: a self-supervised framework for GRN inference. This framework enables pretraining without labels and achieves accurate GRN inference by fine-tuning downstream linear classifiers while maintaining consistency during network updates. SIGMA comprises two core steps: (i) extracting correlation representations during pretraining without labels, and (ii) inferring regulatory relationships for downstream tasks. As illustrated in Figure 1, SIGMA comprises the following two core steps: (i) a pretraining phase that extracts gene correlation representations using an encoder without labels, and (ii) downstream tasks that infer regulatory relationships. Subsequent sections detail each module of this framework.

### 2.1 Self-supervised pretraining

Like existing models, SIGMA aims to explore the interactions between TF-target pairs. However, unlike supervised methods, which rely on labelled data for training, SIGMA uses an unsupervised strategy based on correlations. Specifically, during the pretraining step, SIGMA treats the construction of correlations as a regression problem. For all TF-target pairs, we randomly mask their expression sub-vectors and require the model to reconstruct the whole sequences using the unmasked sub-vectors, while ensuring consistency between the reconstructed and original sequences. SIGMA’s pretraining comprises four modules: sliding window, random masking, encoder, decoder, and predictor. We will discuss each module in detail below.

#### 2.1.1 Window sliding

**FIGURE 2:**
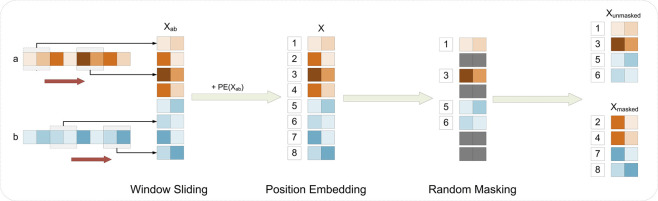
The figure details the construction for entities $X_{\mathrm{unmasked}}$ and $X_{\mathrm{masked}}$ , using genes **(a,b)** as prototypes. It represents the processes of window sliding, position embedding, and random masking.

To represent all gene pairs uniformly, we used a sliding window operation to divide the expression sequences into non-overlapping sub-vectors (window size: s ), as Figure 2, which helped us monitor correlations in expression levels across various developmental stages. Given gene a , the expression vector $G_{a}$ consists of n cells: $G_{a}=\left\{G_{a,1},G_{a,2},\ldots ,G_{a,n}\right\}$ . Each sub-vector $G_{a,l}$ within gene a is defined as $G_{a,l}=\left\{G_{a,l\times s},G_{a,l\times s+1},\ldots ,G_{a,l\times s+\left(s-1\right)}\right\}$ , where l represents the number of patches intercepted by the sliding window (size: s ), and any segment smaller than s at the end is discarded. $X_{ab}$ comprises the patches of TF a and its target b . To retain the temporal and positional context of these sub-vectors, sine-and-cosine positional embedding (PE) (Vaswani et al., 2017) is applied to $X_{ab}$ :

$$X=X_{ab}+PE\left(X_{ab}\right),$$ (1)

$$PE\left(m,2n\right)\;=\;\sin\left(\;\frac{m}{10000^{2n/s}}\;\right),\;PE\left(m,2n+1\right)\;=\;\cos\left(\;\frac{m}{10000^{\left(2n+1\right)/s}}\;\right),$$ (2)

In Equations 1, 2, where m represents the position of each subvector in $X_{ab}$ , 2n represents the even patches, and 2n+1 represents the odd patches.

#### 2.1.2 Random masking

We apply random masking operations to $X_{ab}$ to improve the framework’s ability to identify TF-target correlations. Similar to MAE, we employ a high-ratio masking mechanism (Devlin et al., 2019) to randomly partition the input X into masked $X_{\mathrm{masked}}$ and visible $X_{\mathrm{unmasked}}$ regions. During pretraining, the model’s objective is to reconstruct the prediction $X_{\mathrm{pred}}$ based exclusively on $X_{\mathrm{unmasked}}$ , while preventing information leakage from $X_{\mathrm{masked}}$ and ensuring high feature consistency between $X_{\mathrm{pred}}$ and $X_{\mathrm{masked}}$ in the latent space. Our experiments indicate that a 70% masking rate is optimal.

#### 2.1.3 Encoder

The principle of SIGMA is to decompose GRN inference into two stages: “correlation capture” and “interaction determination”. We introduce pretraining to address the issue of obtaining high-quality training results for models being difficult due to low-quality labels. To capture correlations, we use a multi-layer Transformer as the encoder to extract the relevance representation of $X_{\mathrm{unmasked}}$ . Each encoder layer comprises two submodules: a multi-head self-attention (MHSA) mechanism and a feed-forward network (FFN) (Vaswani et al., 2017). Within the MHSA, the input $X_{\mathrm{input}}$ undergoes linear transformations to generate Query (Q) , Key (K) , and Value (V) :

$$Q=X_{\mathrm{input}}\cdot W_{Q},\;K=X_{\mathrm{input}}\cdot W_{K},\;V=X_{\mathrm{input}}\cdot W_{V},$$ (3)

In Equation 3, where $W_{Q}$ , $W_{K}$ , and $W_{V}$ denote the respective weights, which are then divided into multiple matrices. Each matrix corresponds to a single attention head. For head i , the attention score $Attn_{i}$ is computed as follows:

$$Attn_{i}\left(Q_{i},K_{i},V_{i}\right)=\text{softmax}\left(\frac{Q_{i}K_{i}^{T}}{\sqrt{d_{k}}}\right)V_{i},$$ (4)

In Equation 4, where $d_{k}$ represents the dimension of $K_{i}$ . After computing the scores for h heads, their outputs are concatenated to form a single attention weight $A=\left[Attn_{1},Attn_{2},\ldots ,Attn_{h}\right]$ . This concatenated weight is then linearly transformed by the FFN to generate the output representation.

We use the encoder to obtain separate representations of $X_{\mathrm{unmasked}}$ and $X_{\mathrm{masked}}$ , respectively. It is important to note that these are fed into the encoder independently, rather than concurrently. This design maximises the prevents the encoder from acquiring information from $X_{\mathrm{masked}}$ , thereby enabling it to focus more intently on extracting relevant features from $X_{\mathrm{unmasked}}$ . We then obtain hidden representations $Z_{\mathrm{unmasked}}$ and $Z_{\mathrm{masked}}$ from the input through the encoder. Next, we use these representations for reconstruction and alignment.

#### 2.1.4 Decoder and predictor

Like the encoder, the decoder is also based on the Transformer architecture. The key difference lies in the decoder’s input, which comprises $Z_{\mathrm{unmasked}}$ . We arrange $Z_{\mathrm{unmasked}}$ according to the bit order of the original sequence X , filling any missing positions with mask tokens. The decoder can then infer $Z_{\mathrm{masked}}$ from $Z_{\mathrm{unmasked}}$ , thereby reconstructing the intrinsic relationships within $X_{ab}$ . We denote the decoder’s output as P . We then use a predictor, a linear projection layer, to restore P to $X_{\mathrm{pred}}$ . In addition to making $X_{\mathrm{pred}}$ as similar as possible to the original $X_{\mathrm{masked}}$ , our aim is to minimize the distance between P and $Z_{\mathrm{masked}}$ . To achieve this, we have designed a multi-task optimization as follow.

#### 2.1.5 Multi-task optimization

Our SIGMA uses two pretext tasks: masked representation regression (MRR) (Cheng et al., 2026) and maximizing the similarity of representations (MSR) (Chen and He, 2021). The MRR pretext task aimed to reconstruct the entire $X_{ab}$ . The objective of the predictor’s output, which denoted as $X_{\mathrm{pred}}$ , is to accurately reconstruct the original $X_{\mathrm{masked}}$ . The loss function, based on the mean squared error (MSE), which is defined as follow Equation 5:

$$MSE\left(X_{\mathrm{pred}},X_{\mathrm{masked}}\right)=\frac{1}{n}\sum {\left(X_{\mathrm{pred}}-X_{\mathrm{masked}}\right)}^{2}.$$ (5)

This equation calculates the average squared difference between the actual and predicted gene expression sequences across n data points. By masking and then reconstructing random sub-vectors of the inputs, the MRR captures the correlations between TF-target pairs.

The objective of the MSR is to align P with $Z_{\mathrm{masked}}$ . As the encoder and decoder may produce different representations of the same position, improving the similarity between these representations is essential. This alignment is achieved by minimizing the negative cosine similarity (NCS), expressed as Equation 6:

$$NCS\left(P,\text{sg}\left(Z_{\mathrm{masked}}\right)\right)=-\frac{P}{\|P{\|}_{2}}\cdot \frac{\text{sg}\left(Z_{\mathrm{masked}}\right)}{\|\text{sg}\left(Z_{\mathrm{masked}}\right){\|}_{2}},$$ (6)

where $\|\cdot {\|}_{2}$ is L2-norm, and sg(⋅) denotes the stop-gradient operation (Chen and He, 2021), which treats $Z_{\mathrm{masked}}$ as a constant during gradient calculations. This design prevents information leakage from $Z_{\mathrm{masked}}$ , enabling the encoder to focus solely on $Z_{\mathrm{unmasked}}$ and P , thereby promoting stability.

The overall loss function, D , is defined as Equation 7:

$$D=MSE\left(X_{\mathrm{pred}},X_{\mathrm{masked}}\right)+NCS\left(P,\text{sg}\left(Z_{\mathrm{masked}}\right)\right).$$ (7)

This multi-task approach enables the encoder to accurately identify the correct representation of each sub-vectors. This minimizes the loss of valuable information during reconstruction and ensures similarity between representations.

### 2.2 Downstream inference

During the downstream task, we use the pretrained encoder, which its parameters are frozen, to capture the hidden correlations of the entire $X_{ab}$ . These features are then aggregated via an average pooling layer before being classified by a linear classifier, to determine the presence of interactions. We treat this downstream stage as a binary classification problem, computing the loss using binary cross-entropy (BCE):

$$BCE\;=\;-\frac{1}{N}\;\overset{N}{\underset{i=1}{\sum }}\;y_{i}\log\left(p\left(y_{i}\right)\right)\;+\;\left(1\;-\;y_{i}\right)\log\left(1\;-\;p\left(y_{i}\right)\right),$$ (8)

In Equation 6, where N denotes the total number of samples, $y_{i}$ is the label of the i th sample, and $p\left(y_{i}\right)$ represents its predicted probability, which is constrained within the range (0,1). Our experiments have shown that fine-tuning the encoder while training a linear classifier can improve the model’s performance. However, training only the linear classifier with frozen encoder parameters can also yield the same satisfactory results. Unlike models such as BERT (Devlin et al., 2019), which incorporate an additional class token, our method uses average pooling layers to extract the correlations. We hypothesise that gene interactions are not persistent, but rather occur at specific moments. In GRN inference, pooling layers outperform class tokens in preserving local context, which could enhance inferential performance. This hypothesis will be evaluated in subsequent experiments.

## 3 Experimental results

### 3.1 Datasets

#### 3.1.1 Benchmark datasets

To evaluate the performance of the SIGMA, we used expression profile datasets from seven distinct cell types obtained from the BEELINE project (Pratapa et al., 2020). The cell types included human embryonic stem cells (hESC), human mature hepatocytes (hHEP), mouse dendritic cells (mDC), mouse embryonic stem cells (mESC), mouse erythroid lineage hematopoietic stem cells (mHSC-E), mouse hematopoietic stem cells of the granulocyte-monocyte lineage (mHSC-GM), and mouse lymphoid lineage hematopoietic stem cells (mHSC-L).

Each dataset was supplemented with three types of ground-truth network: STRING networks (Szklarczyk et al., 2019), non-specific network (Liu et al., 2015; Han et al., 2015; Garcia-Alonso et al., 2019), and cell type-specific network (ENCODE Project Consortium, 2012; Xu et al., 2013; Davis et al., 2018; Oki et al., 2018). An additional loss-of-function/gain-of-function (LOF/GOF) ground truth network (Xu et al., 2013) was included specifically for the mESC dataset. We processed the expression profile datasets according to the protocol described in a previous study (Pratapa et al., 2020), focusing exclusively on TF-target interactions. To infer GRNs, we selected the top 500 and 1,000 genes showing the most significant variation, ensuring a corrected p-value of < 0.01 for all TFs. The details of these ground-truth networks for each expression profile are provided in Table 1.

**TABLE 1:** Statistics of expression profiles datasets and ground-truth networks.

Cell types	Cells ^a^	Size	STRING			Non-specific			Specific			LOF/GOF		
​	​	​	TFs^b^	Genes^c^	Regs^d^/Dens^e^	TFs	Genes	Regs/Dens	TFs	Genes	Regs/Dens	TFs	Genes	Regs/Dens
hESC	759	500	343	511	4,257/0.024	283	753	3,441/0.016	34	815	4,545/0.164	—	—	—
		1,000	351	695	5,149/0.021	292	1,138	4,617/0.014	34	1,260	7,084/0.165	—	—	—
hHEP	426	500	409	646	7,523/0.028	322	825	4,129/0.015	30	874	9,939/0.379	—	—	—
		1,000	414	874	9,003/0.024	332	1,217	5,351/0.013	31	1,331	15,558/0.377	—	—	—
mDC	384	500	264	479	4,815/0.038	250	634	3,067/0.019	20	443	756/0.085	—	—	—
		1,000	273	664	5,898/0.032	254	969	3,918/0.016	21	684	1,193/0.082	—	—	—
mESC	422	500	495	638	7,762/0.024	516	890	6,893/0.015	88	977	29,613/0.345	34	774	4,169/0.158
		1,000	499	785	8,479/0.021	522	1,214	8,030/0.013	89	1,385	42,795/0.347	34	1,098	5,742/0.154
mHSC-E	1,072	500	156	291	1,371/0.029	144	442	1,425/0.022	29	691	11,557/0.578	—	—	—
		1,000	161	413	1,826/0.027	147	674	1,960/0.020	33	1,177	21,975/0.566	—	—	—
mHSC-GM	890	500	92	201	748/0.040	82	297	743/0.030	22	618	7,364/0.543	—	—	—
		1,000	100	344	1,311/0.037	88	526	1,358/0.029	23	1,089	14,135/0.565	—	—	—
mHSC-L	848	500	39	70	137/0.048	35	164	279/0.030	16	525	1,398/0.525	—	—	—
		1,000	40	81	154/0.045	37	192	317/0.043	16	640	5,180/0.507	—	—	—

^a^ Number of cells in the gene expression dataset.

^b^ Number of TFs common in the gene expression dataset and standard network.

^c^ Number of genes common to the gene expression dataset and standard network.

^d^ Number of regulations included in standard network.

^e^ Network density calculated as Regs/(TFs∗Genes) .

#### 3.1.2 Breast cancer datasets

We used SIGMA to analyze real-world expression profiles, focusing on the human breast cancer metastasis dataset available in the Gene Expression Omnibus (GEO) database. This database is hosted by the National Center for Biotechnology Information (NCBI) and can be accessed via the identifier GSE123837. This dataset provides insights into the transcriptional and metabolic heterogeneity between primary tumors and the lung metastases. This was obtained using three patient-derived xenograft (PDX) models: HCI001, HCI002, and HCI010. These models represent diverse invasive ductal carcinomas characterized by estrogen receptor (ER), progesterone receptor (PR), human epidermal growth factor receptor 2 (Her2), and basal-like phenotypes.

We integrated regulatory relationships from two comprehensive databases, RegNetwork (Liu et al., 2015) and TRRUST (Han et al., 2015). Preprocessing was essential given the complexity and redundancy of the raw data. We adopted a preprocessing protocol similar to that used for the BEELINE dataset. This involved filtering out genes with low expression detected in fewer than 10% of cells, as well as genes with expression levels and p-values greater than 0.01. The expression levels were then normalized through logarithmic transformation. Using these processed data, we inferred a GRN that included genes common to both the standard network and the gene expression dataset. The detailed statistical data from this analysis are presented in Table 2.

**TABLE 2:** Statistics of breast cancer datasets and their GRNs.

Dataset	Cell types	Cells	TFs	Genes	Regulations	Density
HCI001	Stage IV IDC, npST^a^	247	592	1,256	3,187	0.024
HCI002	Stage IIIA medullary IDC, npST^b^	401	479	921	1,867	0.021
HCI010	Stage IIIC IDC, multi-round chemo^c^	471	583	1,275	3,070	0.028

^a^ Stage IV ER-PR-Her2-basal-like invasive ductal carcinoma with no previous systemic treatment.

^b^ Stage IIIA ER-PR-Her2-basal-like medullary-type invasive ductal carcinoma with no previous systemic treatment.

^c^ Stage IIIC ER-PR-Her2-basal-like invasive ductal carcinoma treated with several rounds of chemotherapies.

### 3.2 Model training and datasets partition

During the pretraining phase, we set the embedding size to 192. For optimization, we used the AdamW optimizer, which was the standard choice for all comparative methods, set the learning rate to 0.001, and maintained all other parameters at their default settings. The batch size was set to 512 for pretraining and then reduced to 128 for the downstream classification. The encoder and decoder both comprised two layers of transformer blocks, each with 12 attention heads and two feedforward networks. We also incorporated the framework’s dropout rate of 0.2. The slice window size was set to 8, with a masking ratio of 70% under the default settings. After 200 epochs of pretraining, we extracted the encoder for use in downstream inference tasks.

GRNs are inherently sparse and characterized by a significant disproportion of positive to negative labels, making the management of imbalanced sample distributions challenging. To ensure a comprehensive performance evaluation, we divided the relationships of all gene pairs within each dataset into training, testing, and validation sets, maintaining a ratio of 3:1:1. This segmentation aimed to preserve the consistency of the positive and negative sample proportions, relative to the original dataset.

SIGMA was developed on the PyTorch platform. All computational experiments, including those for the comparative models, were performed on a system equipped with an Intel Xeon 16,896 k processor, 128 GB of RAM, and eight NVIDIA Tesla V100 GPUs. This setup demonstrates our commitment to leveraging advanced hardware to optimize computational efficiency and model performance.

### 3.3 Evaluating metrics

We treated the task as a binary classification and assigned the label “1” to the TF-target pairs representing “confirmed” interactions, and the label “0” to “unconfirmed” ones.

This study analyzed the expression profiles and constructed time sequences for each TF and their target genes as inputs, obtaining the framework’s output α in the range of $\left(0,1\right)$ . For the binary classification problem, we comprehensively analyzed all output coefficients, set a threshold θ , and classified α≥θ as the positive label, and α<θ as the negative label. Based on these predictions, four possible results are proposed.

- True-Positive (TP): the edge exists in the gold-standard and is predicted by the model.
- True-Negative (TN): the edge does not exist in the gold-standard and is not predicted by the model.
- False-Positive (FP): the edge does not exist in the gold-standard but is predicted to exist by the model.
- False-Negative (FN): the edge exists in the gold-standard, but the model predicts that it does not exist.

Therefore, the precision and recall rates according to these conditions can be calculated as follows:

$$TPR=\frac{TP}{TP+FN},\;FPR=\frac{FP}{FP+TN},\;Recall=\frac{TP}{TP+FN},\;Precision=\frac{TP}{TP+FP}$$ (9)

In Equation 9, the TPR is the proportion of true-positive samples that are correctly identified from the total number of true-positive samples. The FPR is the proportion of false-positive samples detected from the total number of true-negative samples. The recall rate is the proportion of samples that are correctly identified as positive samples. The precision rate is the proportion of samples that are correctly inferred as positive among all samples inferred as positive.

The selection of different thresholds can lead to distinct FPR, TPR, precision, and recall values when inferring GRNs. To evaluate the experimental results, we used 5-fold cross-validation on the GRN inference task, choosing the area under the receiver operating characteristic curve (AUROC) and the area under the precision-recall curve (AUPRC) as our evaluation metrics. The ROC curve was delineated using the FPR and TPR, with the AUROC representing the area beneath it. Similarly, the PR curve was plotted using precision and recall, with the corresponding area defined as AUPRC.

### 3.4 Performance evaluation

#### 3.4.1 Evaluation of SIGMA on benchmark datasets

To evaluate the effectiveness of SIGMA, we compared its performance with state-of-the-art supervised and unsupervised models using benchmark datasets from the BEELINE framework. The evaluated unsupervised methods included GENIE3 (based on a decision tree) and its successor, GRNBoost2 (based on a gradient boosting machine). The supervised methods included DeepSEM, which is based on variational autoencoders; GNE, which utilizes multilayer perceptrons; CNNC, which employs CNN; and DGRNS, which extends CNNC by incorporating RNN with modules for both temporal and spatial learning. Additionally, STGRN uses a transformer architecture similar to ours, focusing directly on supervised GRN inference.

**FIGURE 3:**
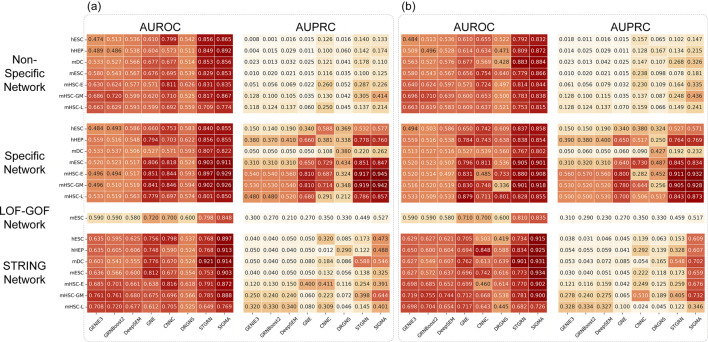
Performance comparison of different models. We conducted GRN inference analyses on seven expression profiles using four well-established ground-truth networks. We divided each dataset into two distinct segments: the top 1,000 highly variable genes and the top 500 highly variable genes, denoted as **(a)** and **(b)**, respectively. The effectiveness of GRN inference in each dataset was assessed by calculating the AUROC and the AUPRC score.

The results are shown in Figure 3. These findings indicate that SIGMA consistently outperforms other methods in terms of AUROC and AUPRC metrics. Unsupervised models tend to emphasize universal correlations between genes and typically use regression methods to ascertain feature importance. However, gene interactions are context-specific and not uniformly present across all the conditions. Furthermore, the efficacy of these models for inference is greatly reduced without labels, resulting in a significant decrease in their performance.

Although supervised models generally perform better, their success depends critically on the reliability of the labels. The presence of false negatives in regulatory labels greatly reduces the accuracy of the GRN inference. Furthermore, models such as CNNC and DGRNS, which convert expression sequences into feature matrices similar to those used in image analysis, tend to retain excessive noise from sequences. This noise has an adverse effect on GRN inference, hindering the understanding of the model.

Our method significantly improves the accuracy of GRN inference, surpassing that of state-of-the-art techniques. It excels at extracting correlation representations from expression profiles and revealing the potential regulatory mechanisms. Moreover, our method can operate without prior labelling, making it ideal for discovering novel GRN subtypes.

#### 3.4.2 Inferring GRNs across subtypes

**FIGURE 4:**
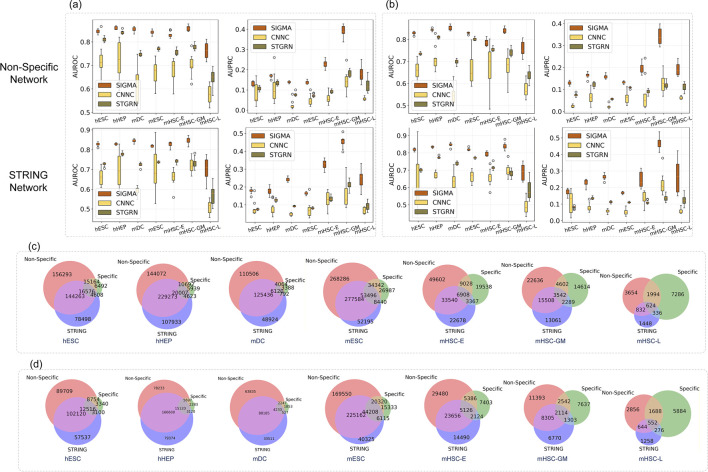
This section evaluates the inferential capabilities of various models within the transfer learning framework. In line with previous studies, we conducted thorough comparisons using two datasets: one comprising the **(a)** top 1,000 highly variable genes and one comprising the **(b)** top 500. **(c)** The Venn diagram shows the overlap of TF-target pairs in each network for the top 1,000 genes in different cell types. **(d)** The Venn diagram illustrates the overlap of TF-target pairs in each network based on the top 500 genes in various cell types.

We used known regulatory patterns to explore the adaptability to previously unseen subtypes, helping us understand new pattern features. We compared SIGMA with two other models: CNNC and STGRN. These models were pretrained on cell-type-specific networks; we then froze the parameters of the backbone and only fine-tuned only the final classification layer to infer non-specific and STRING networks, thereby simulating the conditions of adapting new regulatory patterns. We validated the results using 5-fold cross-validation, as shown in Figures 4a,b. Additionally, we also tested the performance of transferring from pretraining on non-specific networks to inferring cell-type-specific and STRING networks (see Supplementary Figures S1, S2). The outcomes indicate that SIGMA outperforms both CNNC and STGRN, with the latter exhibiting a significant performance decline (compared with Figure 3), demonstrating SIGMA’s superior adaptability in transfer learning with limited retraining.

To investigate this further, we integrated the TF-target pairs from these datasets and constructed Venn diagrams (refer to Figures 4c,d). We observed that, except for mHSC, the cell-type-specific networks for the other cell types contained fewer pairs than the other two networks. However, a significant proportion of these pairs in the cell-type-specific networks were also present in the other two networks. This aligns with the challenges faced when attempting to infer additional unknown GRNs using a limited set of known GRNs. SIGMA not only achieves higher accuracy, it also exhibits reduced variance and fewer anomalies. Notably, SIGMA’s performance was superior in transfer tasks. It remained stable, deviating minimally from the original training outcomes, indicating stronger adaptability and robustness in transfer scenarios rather than true label-free generalization.

The CNNC and STGRN models performed well for the three mHSC cell types shown in Figure 3, but their performance declined significantly in Figure 4. Comparing the Venn diagrams revealed that, unlike other cell types, the cell-type-specific network of the mHSC dataset had a relatively small gap in the number of TF-target pairs between the three networks, but a relatively low proportion of overlap. These extensive non-overlapping pairs can be considered noise that is unrelated to the task. The poor performance of the CNNC and STGRN models suggests that they are more sensitive to noise, resulting in decreased performance. In contrast, SIGMA is less influenced by noise and exhibits greater robustness.

SIGMA’s effectiveness in managing large-scale expression profiles with fewer prior labels is particularly notable. Its ability to extract universal representations significantly enhances its adaptability in transfer tasks. This is a crucial attribute of models aimed at improving the performance of emerging and unseen GRN subtypes. Notably, the experimental setup we adopted aligns with the more stringent evaluation criteria of unseen-TF or unseen-target splits: the cell-type-specific networks used for pretraining contained far fewer genes overlapping with those in the non-specific and STRING networks, effectively simulating the scenario where the model faces unseen regulatory factors or targets in downstream tasks. Under this setting, SIGMA still outperforms CNNC and STGRN without performance degradation, fully demonstrating its superiority.

### 3.5 Ablation experiment

#### 3.5.1 Hyperparameters robustness evaluation

To ascertain the robustness of SIGMA in the context of hyperparameter variations, we conducted a sensitivity analysis. Specifically, we examined how altering the depth and number of attention heads within the encoder and decoder architectures. This enabled us to identify the optimal configuration.

**FIGURE 5:**
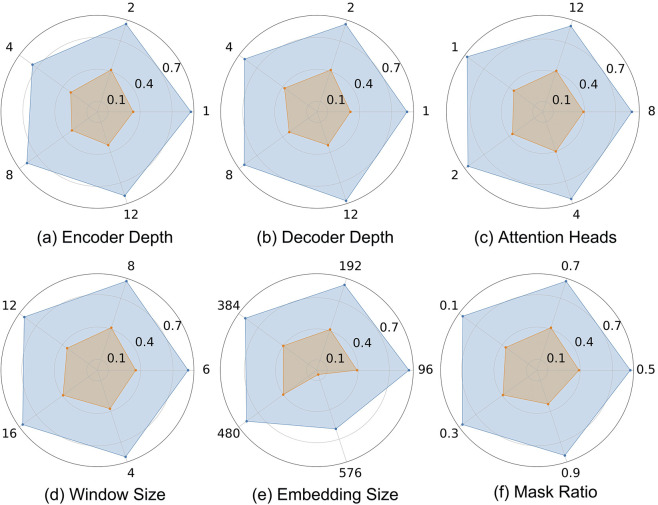
A performance comparison under varying hyperparameters. The blue line shows the AUROC scores for the different configurations, whereas the orange line shows the AUPRC scores. Panels **(a–c)** show the results of the analysis of various model hyperparameters, including the number of layers, number of attention heads, and network depth. Additionally, Panels **(d–f)** show how the outcomes differ under various input conditions, such as changes in the sliding window size and mask ratio. In each case, the hyperparameters were adjusted independently, while all other parameters remained at their baseline values.

We also investigated the effects of variations in the input parameters. These modifications included adjusting the sizes of the sliding windows, embedding size, and mask ratios during the input phase. We used the top-1000 MHSC-GM dataset from the non-specific network as the benchmark. Each hyperparameter was modified individually, with all other parameters kept constant to isolate the effect of each adjustment. The results are shown in Figure 5.

Our findings revealed that increasing the number of attention heads significantly improved the AUPRC scores for both the encoder and decoder. This suggests that additional attention heads enhance the model’s capacity to focus on the relevant features. Conversely, deeper layers did not yield better outcomes, primarily because of the increased likelihood of overfitting. Overfitting occurs when a model treats noise in expression profiles as significant, impairing its generalizability.

We observed that varying the embedding size induces substantial fluctuations in performance (see Figure 5e). This phenomenon can be attributed to the noise in gene expression profiles. The core challenge of GRN inference is to extract genuine regulatory interactions from noisy observations. Projecting expression sequences into high-dimensional spaces amplifies noise propagation, causing effective signals to become further diluted. This finding suggests that certain prevalent practices in computer vision and natural language processing do not readily transfer to gene regulatory network inference. The frequent gradient explosion observed during training further corroborates the model’s instability under specific configurations.

The sensitivity analysis of mask ratios in Figure 5f corroborates this interpretation. At lower mask ratios, the model retains access to sufficient input information, thereby mitigating the impact of noise. Conversely, as the mask ratio increases (90%), the model struggles to discriminate between noise and genuine signals, resulting in performance degradation. However, excessively low mask ratios (10%) hinder the model’s capacity to identify TF-target relationships, thereby limiting learning efficacy. We found that a higher mask ratio, such as We then conducted experiments to systematically evaluate the model’s robustness against noise.

#### 3.5.2 Noise robustness evaluation

**FIGURE 6:**
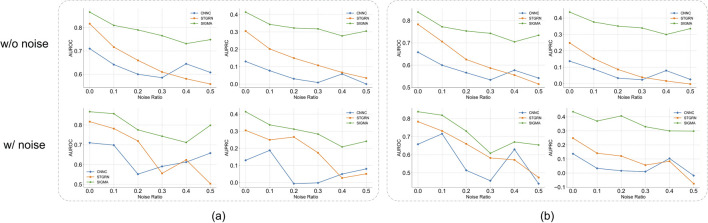
The impact of noise on model performance. This is a comparison of two distinct datasets: **(a)** the top 1,000 and **(b)** the top 500 most highly variable genes. All evaluations were conducted using noisy test data to assess the models’ ability to generalise in noisy conditions.

To evaluate the robustness of each framework to noise, we trained each model using the top-1000 and top-500 mHSC-GM datasets from the non-specific network. Each model was trained under two conditions: “with noise” (w/noise) and “without noise” (w/o noise). During the testing period, all experiments were in a noisy environment. Performance was measured at various noise levels, and the results are shown in Figure 6.

Experimental results demonstrate that SIGMA achieves optimal inference performance in noisy conditions. The model exhibits remarkable noise-resistant characteristics, maintaining high predictive accuracy across varying noise levels—a distinctive advantage not observed in comparable models. This superiority stems from two key design elements. Firstly, during data preprocessing, TF-target pairs are segmented to minimize extraneous noise introduction. Secondly, constructing pretext tasks enables the model to learn genuine regulatory associations despite interference from noise.

It is important to emphasize that GRN inference is fundamentally defined by the high noise levels inherent in gene expression profiles as “extracting true regulatory interactions from noise.” Consequently, noise robustness is a critical metric for evaluating model efficacy. These observations further substantiate the superiority of SIGMA for this task.

#### 3.5.3 Without fine-tune, still robust

**FIGURE 7:**
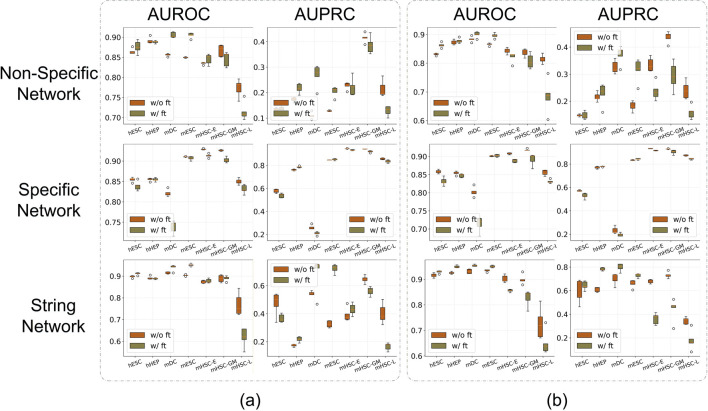
The influence of fine-tuning on the performance of models during downstream inference tasks. This is a comparison of two distinct datasets: **(a)** the top 1,000 and **(b)** the top 500 most highly variable genes. The performance of each model configuration was assessed using a rigorous five-fold cross-validation approach.

To evaluate the effect of fine-tuning on SIGMA’s performance, two configurations were set up: “without fine-tune” (w/o ft) and “with fine-tune” (w/ft) as Figure 7. This distinction highlights the importance of the pretraining phase in improving model accuracy during fine-tuning. Under both setups, SIGMA demonstrated a superior downstream classification performance. However, “with fine-tune” did not always outperform “without fine-tune.” The two configurations often produced comparable results across different expression profiles and network conditions, highlighting the robustness of our pretraining phase and its ability to deliver competitive results without additional fine-tuning.

Furthermore, the “with fine-tune” configuration requires more computational resources and extends the training time, which could affect performance consistency. In scenarios demanding computational efficiency and stability, “without fine-tune” is preferable. It also avoids potential overfitting in sparsely labelled datasets, such as those in non-specific and STRING networks, where label scarcity stems from a large number of unverified interactions. Conversely, in cell-type-specific networks with a higher proportion of verified interactions, the “without fine-tuning” model often yields better results, enhancing consistency and the model’s ability to effectively analyze complex gene expression patterns. This capability is crucial for deciphering the fundamental biological dynamics and identifying potential regulatory mechanisms within gene networks.

#### 3.5.4 Evaluation of the efficacy of modules

**FIGURE 8:**
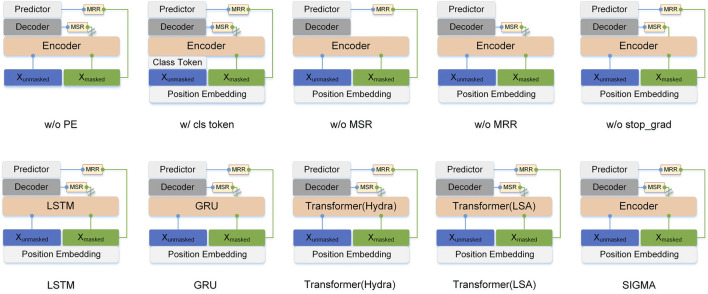
Ablation in SIGMA architecture. We used the addition and cancellation of some modules to explore their impact on performance, and provided their abstract framework.

We conducted module ablation studies to evaluate the impact of various components. We systematically removed each component and assessed its effect on the performance (as shown in Figure 8). During the pretraining step (see Figure 1c), our framework can be summarized as follows:

After applying *PE* to the input, the resulting sub-vectors are randomly partitioned into $X_{\mathrm{unmasked}}$ and $X_{\mathrm{masked}}$ portions. We then employed a vanilla Transformer-based *encoder* and *decoder* are then employed to reconstruct the sequence, and the training process is jointly optimized with *MRR* and *MSR* tasks. The MSR task applies a *stop-gradient* operation to the $X_{\mathrm{masked}}$ portion.

**FIGURE 9:**
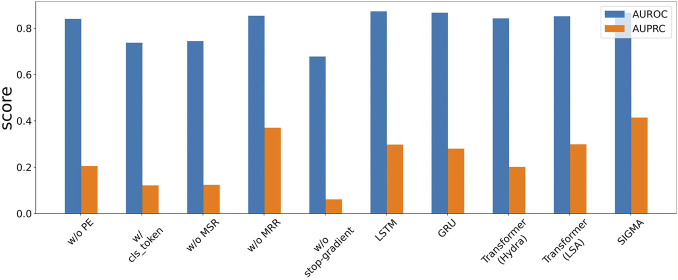
We used bar charts to compare the performance of various module ablations. The ablations were organized into three distinct categories: those affecting the input to the encoder-decoder, those involving the internal structure of the encoder-decoder, and those related to its output. To enable thorough comparison, the AUROC and AUPRC metrics were combined in a single bar chart.

For inputs prior to the encoder-decoder stage, we tested the effects of removing the PE and adding class tokens. At the encoder-decoder stage, we compared the performance of long short-term memory (LSTM) (Hochreiter, 1997) and gated recurrent unit (GRU) (Cho et al., 2014) modules, which are based on RNN. We also compared these with transformer-based models, in which the MHSA was replaced with either the Hydra Attention (Bolya et al., 2022) or Local Self-Attention (LSA) (Lee et al., 2021) mechanism. At the output stage, we ablated the MRR and MSR tasks individually to measure their respective contributions. We also examined the necessity of the stop-gradient operation. The findings of these experiments are shown in Figure 9.

Our experimental results indicate that each component of our model significantly contributes to its overall performance. While prior research (Chen et al., 2021) has demonstrated that PE provides only a minor improvement in other domains, it is crucial for GRN inference in our study. Although the class token is widely used, it does not meet our performance expectations in this context. This shortfall is primarily due to the unique regulatory patterns of expression profiles at different time points, which make feature aggregation using class tokens challenging to achieve. Using a simple average pooling method yielded superior results.

These findings highlight the impracticality of applying image recognition methods to GRN inference. When validating the backbone, the vanilla transformer achieved the best results. Despite the existence of many optimized transformer-based models, the efficacy of the vanilla model remains unparalleled.

Our experiments revealed that the best results were achieved only when both the MRR and MSR tasks were optimized simultaneously at the output stage. In addition, we demonstrated the necessity of the stop-gradient operation. Together, these modules form SIGMA, enabling it to achieve optimal overall performance.

### 3.6 Investigation of SIGMA’s predictions

We further investigated the potential applicability of SIGMA inference in studying human diseases further, using breast cancer as a case study. The GRNs were constructed based on the top- k predictions from SIGMA, where k corresponds to the number of edges in the gold-standard network (set at 500).

**FIGURE 10:**
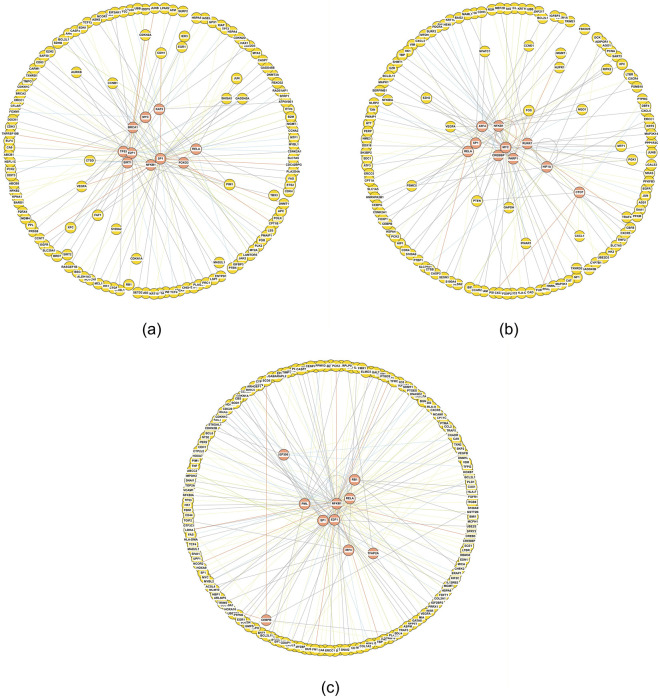
Visualization of the predictions: **(a–c)** show the predicted GRNs derived from the HCI001, HCI002, and HCI010 datasets, respectively, using the SIGMA. The red and yellow nodes represent TFs and their target genes, respectively. The interactions in the established gold standard networks are depicted with black edges. Red, blue, and green edges signify predicted interactions confirmed in non-specific, cell-type-specific, and STRING networks, respectively. Yellow edges represent interactions that are unique to the gold-standard network that have not been identified in the aforementioned networks.

We focused our analysis on the ten TFs with the highest degree and visualized their GRNs, as depicted in Figure 10. Our reconstructed network exhibited hierarchical and scale-free topological properties consistent with classical GRN architectures. SIGMA successfully recovered interactions documented in the gold-standard networks (indicated by black edges in Figure 10a) and identified established hub regulators, including SP1 and E2F1. Beyond these validated interactions, we examined edges absent from the gold-standard network. These novel predictions were then compared against three complementary data sources: non-specific, cell-type-specific and STRING networks derived from BEELINE datasets. Some of these edges were supported by one or more BEELINE networks, suggesting potential biological relevance (Figure 10a). Specifically, edges not present in the gold-standard networks but appearing in non-specific networks are marked in red, edges supported by cell-type-specific networks are marked in blue; and edges corroborated by STRING networks are marked in green. While these computational consistencies do not constitute experimental validation, they suggest that SIGMA predictions are consistent with existing biological knowledge and warrant further experimental investigation. We emphasise that the absence of interactions in current databases does not necessarily imply incorrect inference. However, independent experimental validation remains essential for establishing biological significance.

In terms of hub genes, SP1 and E2F1 were identified as core regulators in all three networks, emphasising their pivotal and ubiquitous role in breast cancer regulatory networks. However, subtle differences were observed in the secondary hub genes. For example, the PDX model representing early-stage breast cancer showed increased connectivity of TP53, while the model representing advanced-stage disease displayed an abundance of regulatory edges associated with MYC. Additionally, black edges were predominantly involved in core cell cycle regulation and cell proliferation pathways, which are fundamental to all breast cancer phenotypes. In contrast, the top edges specific to each model varied distinctly: edges supported by non-specific networks (red edges) were more prevalent in the triple-negative breast cancer PDX model, suggesting the presence of distinct, non-canonical regulatory mechanisms in this subtype. Edges supported by cell-type-specific networks (blue edges) were enriched in the hormone receptor-positive model, reflecting the presence of subtype-specific regulatory signatures. Edges corroborated by STRING networks (green edges) showed a higher degree of overlap in the metastatic PDX model, suggesting the presence of conserved, disease-related regulatory pathways involving protein-protein interactions in advanced disease.

To evaluate the functional coherence of the predicted networks, we performed Gene Ontology (GO) enrichment analysis (Bauer et al., 2008) for the ten most connected TFs across Biological Process, Cellular Component, and Molecular Function categories, alongside KEGG pathway enrichment analysis (Kanehisa and Goto, 2000). High-confidence results were retained (p−value<0.05) and summarized the top 10 GO and KEGG terms were summarized (as shown in Supplementary Figure S3). In the HCI001 dataset, seven of the top ten GO terms in the biological process category are supported by prior literature in breast cancer contexts, while the remaining three represent hypotheses for future investigation. Preliminary comparative analysis of the GO terms associated with the top TFs in each network showed that the early-stage model was enriched with terms related to cell differentiation and DNA damage repair; the advanced-stage model with terms linked to cell invasion and metastasis; and the subtype-specific model with terms involved in immune microenvironment regulation. These conserved and divergent features confirm SIGMA’s ability to capture context-specific regulatory patterns and provide valuable insights into the molecular mechanisms of breast cancer depending on stage and phenotype. These findings suggest that SIGMA-derived networks can generate biologically plausible hypotheses to inform subsequent experimental and therapeutic studies, pending orthogonal validation.

## 4 Conclusion

We developed a self-supervised learning framework, called SIGMA, to infer GRNs from gene expression profiles. It addresses several challenges in GRN inference, such as unreliable label quality, variability in results due to model updates, and the need for improved model transferability. By incorporating pretasks that capture the intrinsic association features of TF-target pairs, SIGMA significantly enhances performance. This method not only obviates the need for high-quality labels but also ensures consistent results across the model iterations.

Our comprehensive analysis used the BEELINE benchmark datasets, encompassing various species, lineages, and network sizes, and included both unsupervised and supervised methods. SIGMA was consistently more effective than the other approaches in all tested scenarios, demonstrating its ability to analyze gene expression profiles and elucidate potential regulatory interactions.

We applied the SIGMA algorithm to investigate the GRN associated with breast cancer. Focusing on the top 10 TFs with a high density of regulatory connections, we organized these interactions into a hierarchical, scale-free network architecture. This approach identified key genes that served as network hubs. Our study also revealed novel regulatory relationships that were subsequently validated through comparative analysis. GO and KEGG pathway enrichment analyses revealed biologically relevant terms related to breast cancer treatment. These results demonstrate SIGMA’s ability to retrieve established gene interactions and identify previously unknown regulatory genes and patterns. This could deepen our understanding of the biological mechanisms underlying breast cancer and accelerate the development of drugs.

However, the current model still cannot bypass the limitation that “unconfirmed” labels are judged as negative, and the SIGMA we proposed only serves as a mitigation solution. In the future, we will focus on this issue and attempt to solve it.

## Data Availability

Publicly available datasets were analyzed in this study. This data can be found here: https://doi.org/10.5281/zenodo.3378975.
