# Supplementary material for: SIGMA: self-supervised inference of gene networks via masked auto-encoding
Source: Front Genet. 2026 Jun 5;17:1825728. doi: 10.3389/fgene.2026.1825728 (PMC13278733; doi:10.3389/fgene.2026.1825728)
Supplement: Supplementary file 1 [file DataSheet1.pdf]

1

Supplementary Material for SIGMA: Self-supervised Inference of Gene Networks via Masked Auto-encoding

Qian Wang [1], **Ziyi Zhang**[2], **Nan-Qing Liao**[3,4], **Shibin Yang**[5,6,*], **Zehua He** [4,6,*]

[1] *School of Medicine, Guangxi University, Nanning, 530004, China*
[2] *Department of Pulmonary and Critical Care Medicine, The First Affiliated Hospital of Guangxi Medical University, No. 6 Shuangyong Road, Nanning, Guangxi Zhuang Autonomous Region, 530021, China*
[3] *Department of Plastic Surgery and Burns, Affiliated Hospital of Zunyi Medical University, Zunyi, 563006, China*
[4] *College of Life Science and Technology, Guangxi University, Nanning, 530004, China*
[5] *Department of Gastrointestinal Surgery, The First Affiliated Hospital, Sun Yat-sen University, Guangzhou, 510080, China*
[6] *Department of General Surgery, Guangxi Hospital Division of The First Affiliated Hospital of Sun Yat-sen University, Nanning, 530022, China*

Correspondence*:
Shibin Yang, Zehua He
yangshb@mail.sysu.edu.cn, hezehua2000@126.com

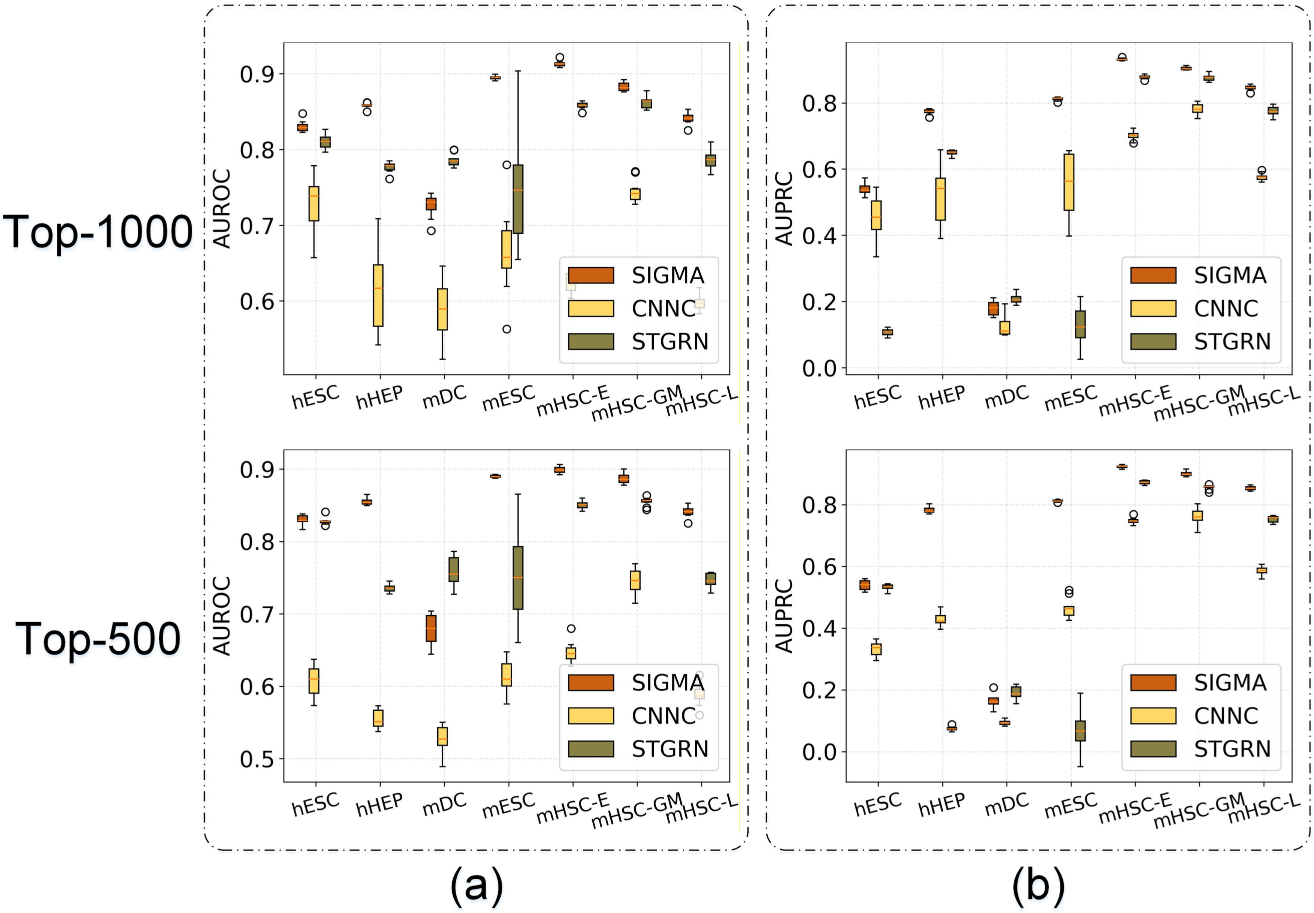

Figure 1. We pretrain on top-1000 and top-500 non-specific networks and use the encoder to infer cell-type-specific networks, and calculate their (a) AUROC and (b) AUPRC scores.

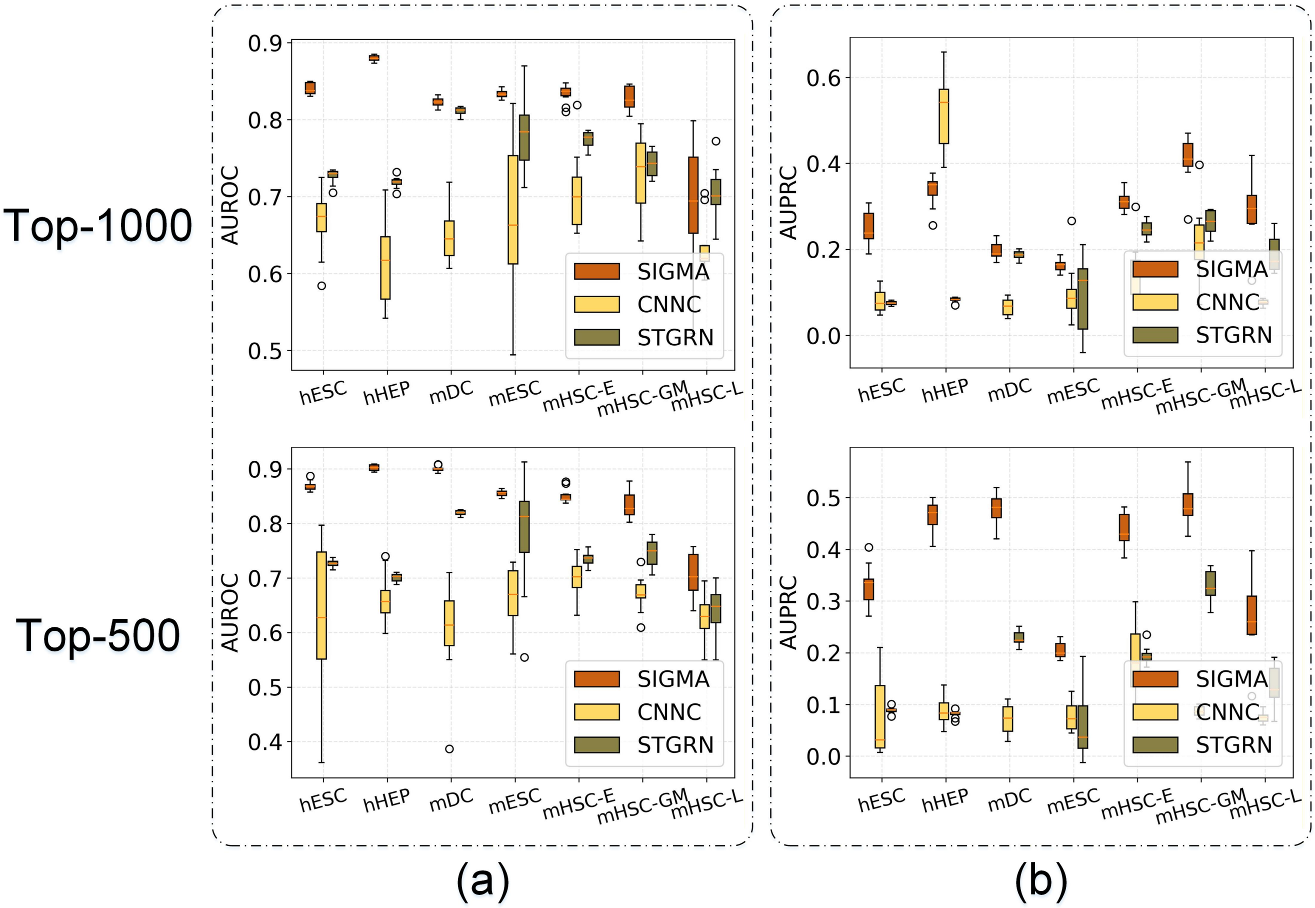

Figure 2. We pretrain on top-1000 and top-500 non-specific networks and use the encoder to infer STRING networks, and calculate their (a) AUROC and (b) AUPRC scores.

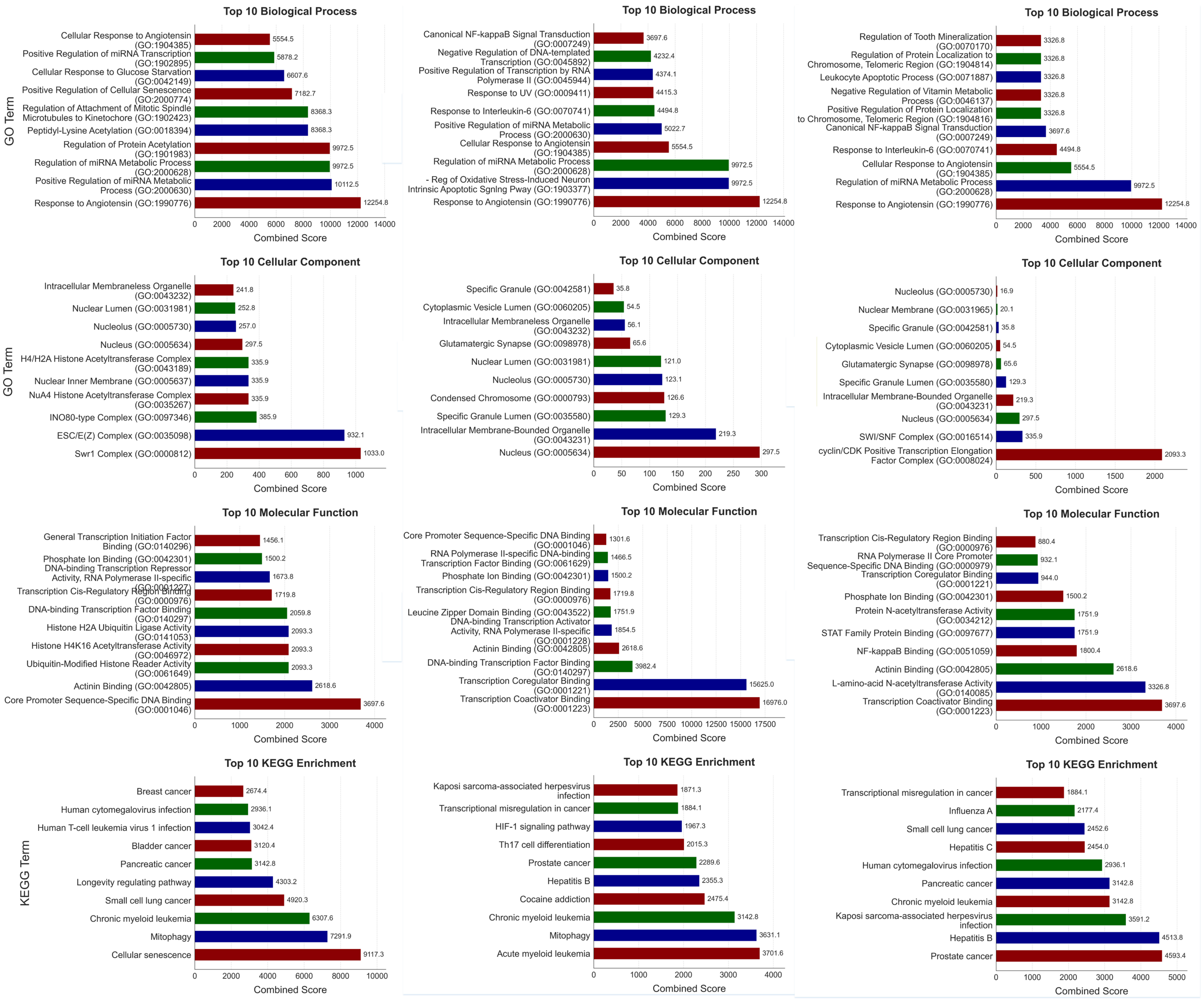

(a) (b) (c)

Figure 3. Enrichment analyses were conducted for GO terms and KEGG pathways associated with specific transcription factors within the predicted networks of (a) HCI001, (b) HCI002, and (c) HCI010. The factors were examined across the GO categories of Biological Process, Cellular Component, and Molecular Function. The analysis focused on terms with p-values below the significance threshold of 0.05. The terms were prioritized based on their level of enrichment, with the top ten terms from each category chosen for a detailed review. This methodology was consistent for both GO and KEGG pathway enrichment assessments.
